# Editorial: Targeting key cellular signaling network for cancer chemotherapy and immunotherapy

**DOI:** 10.3389/fimmu.2024.1425261

**Published:** 2024-05-16

**Authors:** Hao Chi, Lai Jiang, Shengke Zhang, Yunfei Liu, Guanhu Yang, Gang Tian

**Affiliations:** ^1^Clinical Medical College, Southwest Medical University, Luzhou, China; ^2^Department of General, Visceral, and Transplant Surgery, Ludwig-Maximilians-University Munich, Munich, Germany; ^3^Department of Specialty Medicine, Ohio University, Athens, OH, United States; ^4^Department of Clinical Laboratory Medicine, The Affiliated Hospital of Southwest Medical University, Luzhou, China; ^5^Sichuan Province Engineering Technology Research Center of Molecular Diagnosis of Clinical Diseases, Luzhou, China; ^6^Molecular Diagnosis of Clinical Diseases Key Laboratory of Luzhou, Sichuan, China

**Keywords:** cellular signaling network, cancer, chemotherapy, immunotherapy, tumor microenvironment (TME)

## 1 Introduction

Cancer pathology is diversely complex, with the key lying in the deep understanding and strategic manipulation of cellular signaling networks (1). In this Research Topic of the journal on “Targeting Key Cellular Signaling Networks for Cancer Chemotherapy and Immunotherapy,” 16 cutting edge articles have been accommodated. Most of these articles not only bring forth the enormous therapeutic potential of precisely targeting these pathways but also provide a deeper understanding of the various signaling networks and emphasize upon highly innovative strategies of combining chemotherapy with immunotherapy. Some of these strategies promise not only to enhance therapeutic efficacy but also to reduce cancer’s resistance to current treatments. We are at the cusp of a new dawn in the understanding of oncology, a time at which precision medicine is just about to come of age (2, 3). The articles presented in this Research Topic contain pathbreaking perspectives and futuristic approaches that might change the course of future cancer treatment. The presented research ranges from fundamental biochemical insights to translational and clinical applications, pushing us to challenge the disease with more precision and fewer side effects.

This editorial aims to shed light on this theme by summarizing and discussing some of the key articles in this Research Topic. These articles delve into various aspects including the interaction between TME and immunotherapy, the interaction between immune cell dynamics and immunotherapy, new chemotherapy and therapeutic strategies, and molecular and cellular level mechanisms.

## 2 Interactions between the tumor microenvironment and immunotherapy

Recent studies on crosstalk between the tumor microenvironment (TME) and immunotherapy mainly focus on strategies that potentiate the effect of immunotherapies by decoding complex signaling networks within TME (4). The tumor microenvironment presents itself as a more sophisticated system involving tumor cells, immune cells, fibroblasts, vascular cells, and the extracellular matrix. All these parts interact through cytokines, growth factors, and chemokines, promoting tumor growth, invasion, and metastasis (4). Yang et al. explained how M2 macrophages interfered with the anti-tumor effects of lenvatinib in intrahepatic cholangiocarcinoma. M2 macrophages promote tumor angiogenesis and progress by modulating immune responses and secreting factors that support tumor growth, reducing lenvatinib-induced apoptosis in cholangiocarcinoma cells, while M1 macrophages enhance apoptosis. Zhang et al. provided a thorough study into the molecular landscape complexity of thymic epithelial tumors and discussed the importance of understanding this network to enhance the accuracy of diagnosis and refinement of prognosis evaluation and targeted therapy development. He et al. constructed a disulfide apoptosis-based personalized prognostic assessment in lung adenocarcinoma by integrating single-cell technologies with large-scale RNA sequencing. Ding et al. showed that the ceramide-associated genes have a prognostic potential in melanoma through single-cell sequencing analysis and also highlighted its guiding effect on immunotherapy. Lin et al. combined single-cell RNA sequencing and large-scale RNA sequencing to profile subpopulations of cells within cervical tumors and provide insights into the development of new targetable treatment strategies. The work of Wang et al. pointed out Notch signaling as the key player in the central hub of tumor immunity, hence suggesting a potential target for cancer therapy. Finally, Zhou et al. explored the subpopulations and dynamics of myofibroblasts in clear cell renal cell carcinoma, providing new insights into the interactions between tumor cells and highlighting the potential therapeutic targets. These studies demonstrate how the tumor microenvironment influences disease progression and treatment responses through its complex molecular and cellular dynamics, offering valuable insights for developing more effective treatment strategies.

## 3 Interactions between immune cell dynamics and immunotherapy

Another important field of contemporary study is the dynamics of interactions between immune cells and immunotherapy in cancer treatment (5). It delves into the dynamics of how immune cells regulate and take part in immune responses occurring within the body, and enhancing the activity of those immune cells increases the effect of immunotherapies. It is manifested through direct cytotoxic actions, cytokine production, and interactions with other immune cells playing a critical pivot role in the fight against cancer (6). Effective activation or modulation of these functions of cells can bring out a marked improvement in the efficacy of immunotherapies but also brings numerous challenges like immune escape and resistance to therapy. Fan et al. presented in their paper a discussion about the NK cell-based immunotherapy and its place among the modern methods of ovarian cancer treatment. In this review, the authors summarized the mechanisms by which NK cells can execute antitumor effects and reviewed the challenges and potential of various immunotherapeutic strategies to augment NK cell activity. Song et al. revealed the association between the novel biomarker TBC1D1 and immunotherapy resistance in gliomas, particularly noting that elevated expression of TBC1D1 in macrophages correlates with weakened T-cell function, impaired immune responses, and poor prognosis. These findings suggest that inhibiting TBC1D1 could enhance the effectiveness of immunotherapies, offering a potential new strategy for treating gliomas resistant to current immunotherapies. Collectively, these papers shed light on the importance of immune cell dynamics in effective cancer treatment and open doors for the possible optimization of therapeutic regimens based on targeting specific molecules and cell types.

## 4 New chemotherapy and treatment strategies

The field of cancer treatment is continually developing and optimizing new strategies aimed at enhancing efficacy, reducing side effects, and improving patient survival through precision medicine. With a deeper understanding of cancer biology and advances in technology, researchers design personalized treatment plans based on the specific molecular characteristics of tumors and individual patient differences. These strategies range from traditional chemotherapy to the latest immune and cell therapies. For example, Zhang et al. developed a scoring system of aging risk (SRRS) using aging-related genes for colorectal cancer. As expected, this new tool not only helps predict treatment outcomes but also proves imperative in a series of personalized strategies for immunotherapy. Similarly, in the treatment of hepatocellular carcinoma, Su et al. compared the effects of external beam radiation therapy (EBRT) and transarterial chemoembolization (TACE), providing empirical evidence for choosing the appropriate local treatment method. Furthermore, bibliometric analysis by Huang et al. for neoadjuvant chemotherapy on bladder cancer revealed trends and existing research hotspots in the field, underlining the importance of integrated immunotherapy strategies. In the treatment of peripheral T-cell lymphoma, Fulati et al. assessed the effectiveness of autologous stem cell transplantation (ASCT) as a consolidation therapy, particularly noting significant improvements in efficacy following the use of pegylated liposomal doxorubicin. These case studies reflect how a deep exploration and utilization of molecular and cellular mechanisms in cancer can effectively drive the innovation and optimization of treatment strategies. This not only enhances our understanding of the complexities of tumor therapy but also provides new directions for future treatments.

## 5 Molecular and cellular level mechanisms

In the field of tumor biology, a deep understanding of molecular and cellular mechanisms is crucial for developing effective cancer treatment strategies (7). These mechanisms are not only key to tumor growth and spread but also significantly influence the immune system’s response and the success of therapeutic approaches. With advances in technology, researchers are now able to explore these complex signaling networks in greater detail, thereby advancing personalized medicine. Wang et al. recently carried out an extensive review of the multiple roles of CD24 going beyond that in cancer. They defined important functions of CD24 in different biological processes and diseases, with special emphasis on its role in immune regulation, the cancer immune microenvironment, and targeting it as a therapeutic candidate in autoimmune diseases. Chen et al. focused on the role of the tyrosine phosphatase PTPN11/SHP2 in solid tumors, highlighting its potential as a therapeutic target. Considering its dual role as both a tumor promoter and, in less common instances, a tumor suppressor, targeting SHP2 opens new directions for cancer therapy. In addition, Rong et al. conducted an analysis of the relationship of the prognostic markers associated with oxidative stress and the immune landscape, drug response, and prognosis in the case of skin cutaneous melanoma (SKCM), once again confirming the relevance of oxidative stress in the regulation of the tumor microenvironment and effects of treatment. These studies collectively demonstrate how precise dissection of cancer’s molecular and cellular mechanisms can provide a scientific basis for developing new treatment methods, while also emphasizing the necessity of continued research to advance cancer treatment.

## 6 Conclusion and outlook

Overall, the research featured in this Research Topic highlights the central role of signaling networks in cancer treatment, particularly in the application of chemotherapy and immunotherapy. By delving into the key signaling pathways involved in tumor development, immune regulation networks, and the interactions within the tumor microenvironment, these studies provide critical insights for developing customized treatment strategies tailored to individual patients. It is important to note that clinical and biological validation of these research findings is crucial for their successful translation into practical treatment strategies. Therefore, future work should include further validation studies to confirm the actual efficacy and application potential of these findings.

## Author contributions

HC: Conceptualization, Writing – original draft, Writing – review & editing. LJ: Writing – original draft. SZ: Writing – original draft. YL: Writing – original draft. GY: Writing – review & editing. GT: Writing – review & editing.
